# Phenotype and genetic variation analysis of primary congenital lymphedema caused by FLT4 gene mutations in a fetus

**DOI:** 10.3389/fgene.2025.1700635

**Published:** 2025-11-17

**Authors:** Shanshan Zhai, Limin Yuan, Jing Li, Ling Liu, Lulu Hu, Yanjie Ban, Xuewen Yang, Jie Yang, Xuezhe Ouyang, Nana Huang, Yuan Tian, Ting Wang, Xiao Kong

**Affiliations:** 1 Medical Genetics and Prenatal Diagnosis Department, The Third Affiliated Hospital of Zhengzhou University, Zhengzhou, Henan, China; 2 Obstetric Department, The Third Affiliated Hospital of Zhengzhou University, Zhengzhou, Henan, China

**Keywords:** FLT4 gene, primary congenital lymphedema, fetus, prenatal diagnosis, imaging phenotype, genetic variation

## Abstract

**Introduction:**

The purpose of this retrospective study was to investigate the imaging phenotype and genetic variation of three fetuses with FLT4 gene mutations in three families.

**Method:**

Ultrasound images of the three affected fetuses were collected; fetal specimens were obtained by amniocentesis, and peripheral blood samples were collected from both parents for whole-exome sequencing (WES) and copy number variation sequencing (CNV-seq).

**Result:**

The primary prenatal imaging phenotype of fetuses from three families showed bilateral lower limb and foot dorsum lymphedema. Genetic testing identified a paternal c.3122G>A (p.R1041Q) heterozygous variant of the FLT4 gene (NM_182925.5) in the family 1 fetus, a paternal c.3284G>A (p.S1095N) heterozygous variant of the FLT4 gene (NM_182925.5) in the family 2 fetus, and a maternal c.2560G>A (p.G854S) heterozygous variant of the FLT4 gene (NM_182925.5) in the family 3 fetus. Postpartum follow-up: the fetus in family 1 mainly presented with bilateral foot dorsum lymphedema at birth, which improved significantly after surgical treatment at 1 month of age; the fetus in family 2 had mild lymphedema in both feet at birth, which significantly subsided at 18 months of age; the pregnancy was terminated in family 3.

**Discussion:**

Bilateral lower limb lymphedema is a typical clinical manifestation during the fetal stage. Some cases follow a benign course of natural regression, whereas some cases achieve a good prognosis via surgical intervention. The FLT4 c.3284G>A variant identified in this study has not been previously reported, which broadens the mutation spectrum of this gene. In this study, we provide valuable insights for prenatal diagnosis, genetic counseling, and prognosis evaluation of the disease.

## 1 Introduction

Primary congenital lymphedema (PCL) is a chronic disease caused by the lymphatic system developmental abnormalities or dysfunction, with an estimated incidence of 1/6,000 (Gezginc et al., 2012). The current understanding on the phenotype, variant type, and disease prognosis of fetal-onset lymphedema caused by *FLT4* mutations remains limited. Although it has been found that certain *FLT4* variants are associated with fetal lymphedema, the imaging phenotype variability, family inheritance patterns, and postnatal disease progression caused by variants in different sites remain unclear. Therefore, accurately identifying *FLT4* gene variants and corresponding phenotypes is essential for precise prenatal diagnosis, genetic counseling, and clinical intervention.

## 2 Materials and methods

### 2.1 Subjects

Family 1: a 32-year-old pregnant woman, gravida 1, para 0, visited our hospital at 22 weeks of gestation (WG) because “ultrasound images showed thickening of the subcutaneous tissues in both lower legs and feet of the fetus.” The mother conceived naturally, with normal nuchal translucency (NT) in early pregnancy; the father had bilateral foot dorsum edema at birth, but without medical intervention, it gradually improved with age, and the edema symptoms disappeared at the age of 5 years. The grandfather and grandmother of the fetus had passed away, making it impossible to collect clinical information from them.

Family 2: a 24-year-old pregnant woman, gravida 3, para 1, visited our hospital at 24 WG because “ultrasound images showed thickening of the subcutaneous tissues in both lower legs and feet of the fetus.” The mother conceived naturally, with normal NT in early pregnancy; the father has had bilateral foot dorsum thickening and edema since childhood, and his grandfather and his paternal aunt all have unilateral foot dorsum thickening and edema. The couple has a 5-year-old daughter with left foot edema and spoon-shaped nails.

Family 3: a 27-year-old pregnant woman, gravida 2, para 0, visited our hospital at 23 WG because “ultrasound images showed thickening of the subcutaneous tissues in both lower legs and feet of the fetus.” The mother conceived naturally, with normal NT in early pregnancy; there was no abnormal phenotype identified in the father, and there was no abnormal clinical phenotype identified in the mother and the maternal grandparents.

The comprehensive etiological investigation for fetal edema was performed on all three families after the hospital admission. A level IV fetal ultrasound examination was conducted. Amniocentesis was performed to obtain fetal specimens, and the peripheral blood samples were also collected from both parents for whole-exome sequencing (WES) and copy number variation sequencing (CNV seq).

#### 2.1.1 Ethical approval/patient consent

This study has been reviewed and approved by the Ethics Committee of our hospital (2025-104-01), and both parents have signed the informed consent forms for the testing and clinical research.

### 2.2 Methods

#### 2.2.1 Level IV fetal ultrasound examination

The pregnant woman was positioned supine and underwent systematic screening of the various fetal system structures and the biological diameter measurement, using a Samsung W10 color Doppler ultrasound diagnostic device, with a RAB4-8D volume probe (frequency of 4–8 MHz) and a CA2-9A probe (frequency of 2–9 MHz), under obstetric conditions.

#### 2.2.2 Specimen collection

Ultrasound-guided amniocentesis was performed to extract 20 mL of amniotic fluid from fetuses of the three families. In addition, 2 mL of venous blood samples were collected from both parents of the three families, and then anticoagulated with EDTA. Cellular genomic DNA was extracted using the QIAamp® DNA Blood Mini Kit (250), strictly following the instructions of the kit. Then, an appropriate amount of DNA was collected for quantitative and purity testing using a UV spectrophotometer.

#### 2.2.3 Copy number variation sequencing

The library was constructed following the instructions of the chromosome copy number variation detection kit (reversible terminator sequencing), and the copy number variations (CNVs) were detected using the NextSeqCN500 (Illumina, United States) sequencer. The data were analyzed using the CNV detection algorithm developed by Tattini et al., with detected CNVs with a resolution of more than 100 kb.

#### 2.2.4 Whole-exome sequencing

The library was constructed and quantified following the instructions of the human rare disease-related multi-gene joint detection kit (reversible terminator sequencing) and the KAPA Library Quantification Kit. High-throughput sequencing was performed using the Illumina Novaseq6000 platform (Illumina, San Diego, USA), and the raw data were processed using CASAVA v1.82. More than 85% of the bases met the standard of Q30 or above, whereas more than 95% of the bases met the standard of Q20 or above. The duplication rate did not exceed 30%, and the average sequencing depth of each sample was more than 70X. Sequencing data analysis was conducted using the Verita Trekker® mutation site detection system and GATK (https://software.broadinstitute.org/gatk/). Variant annotation databases mainly included population databases, prediction algorithms, and disease and phenotype databases, such as OMIM (http://www.omim.org), ClinVar (http://www.ncbi.nlm.nih.gov/clinvar), HGMD (http://www.hgmd.org), and HPO (https://hpo.jax.org/app/). The interpretation of the report was based on the American College of Medical Genetics and Genomics (ACMG) guidelines for the interpretation of genetic variations, which classify mutations into five categories: pathogenic (P), likely pathogenic (LP), variant of uncertain significance (VUS), likely benign, and benign. Furthermore, the identified positive variant locus was verified within the family using Sanger sequencing.

## 3 Results

### 3.1 Fetal ultrasound imaging characteristics during pregnancy

**FIGURE 1:**
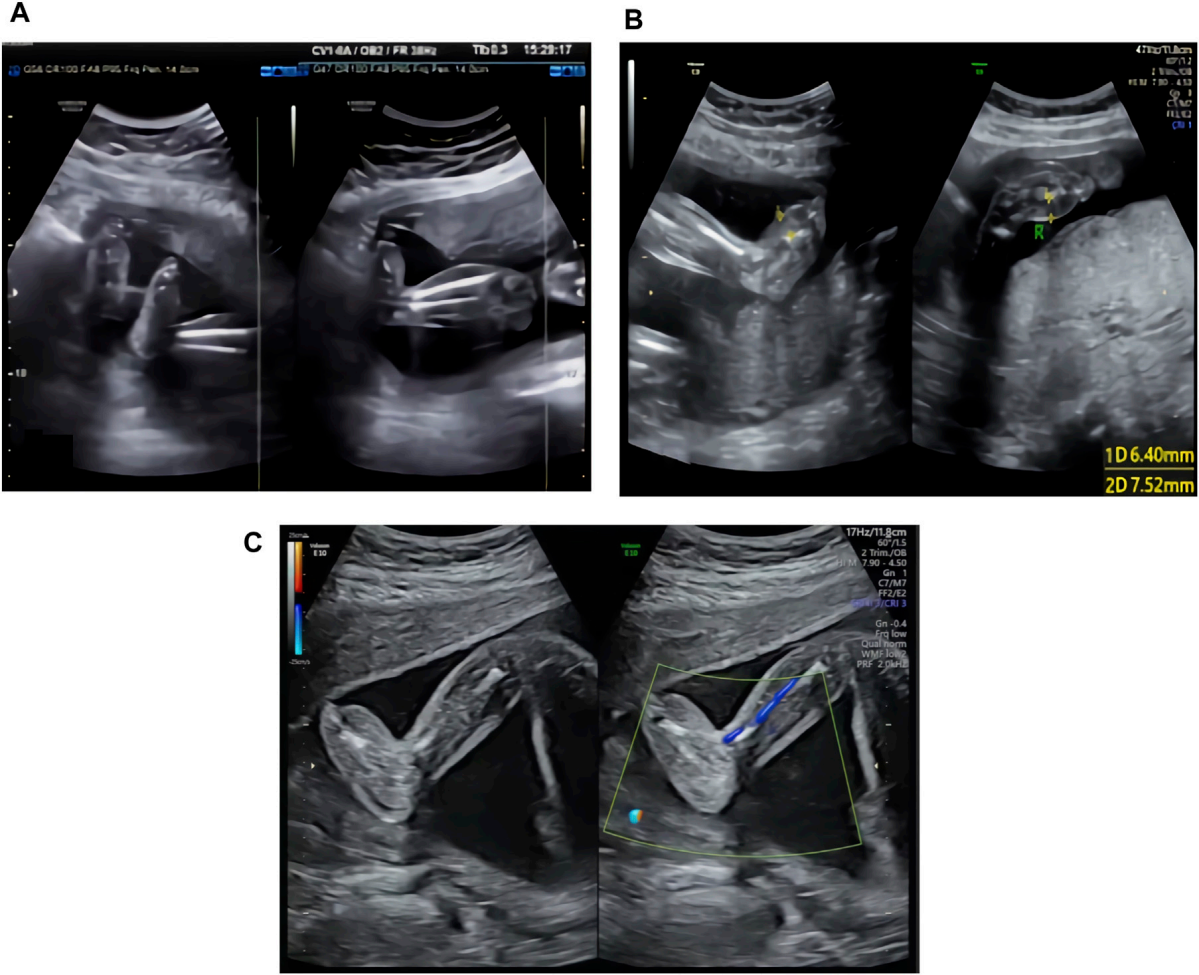
**(A)** Ultrasound image of the family 1 fetus at 22 WG: thickening of the subcutaneous tissues in both lower legs and feet. **(B)** Ultrasound image of the family 2 fetus at 24 WG: edema in both feet and foot dorsum skin thickening. **(C)** Ultrasound image of the family 3 fetus at 23 WG: thickening of subcutaneous tissues in both feet.

The ultrasound examination of the family 1 fetus at 22 WG in our hospital showed thickening of subcutaneous tissues in both lower legs and feet, with thicker areas of approximately 3.9 mm (see Figure 1A).

The ultrasound examination of the family 2 fetus at 24 WG in our hospital showed edema of both feet and thickening of the foot dorsum skin, with thicker areas of approximately 6.4 mm (right side) and 7.5 mm (left side) (see Figure 1B).

The ultrasound examination of the family 3 fetus at 23 WG in our hospital showed thickening of subcutaneous tissues in both feet (see Figure 1C), with thicker areas in the dorsum of approximately 6.1 mm (right side) and 5.4 mm (left side) and in planta pedis of approximately 5.0 mm (right side) and 4.5 mm (left side).

### 3.2 CNV-seq results

No chromosomal aneuploidy or known pathogenic gene CNVs with a resolution of above 100 kb were detected in any of the three families.

### 3.3 WES and Sanger sequencing results

**FIGURE 2:**
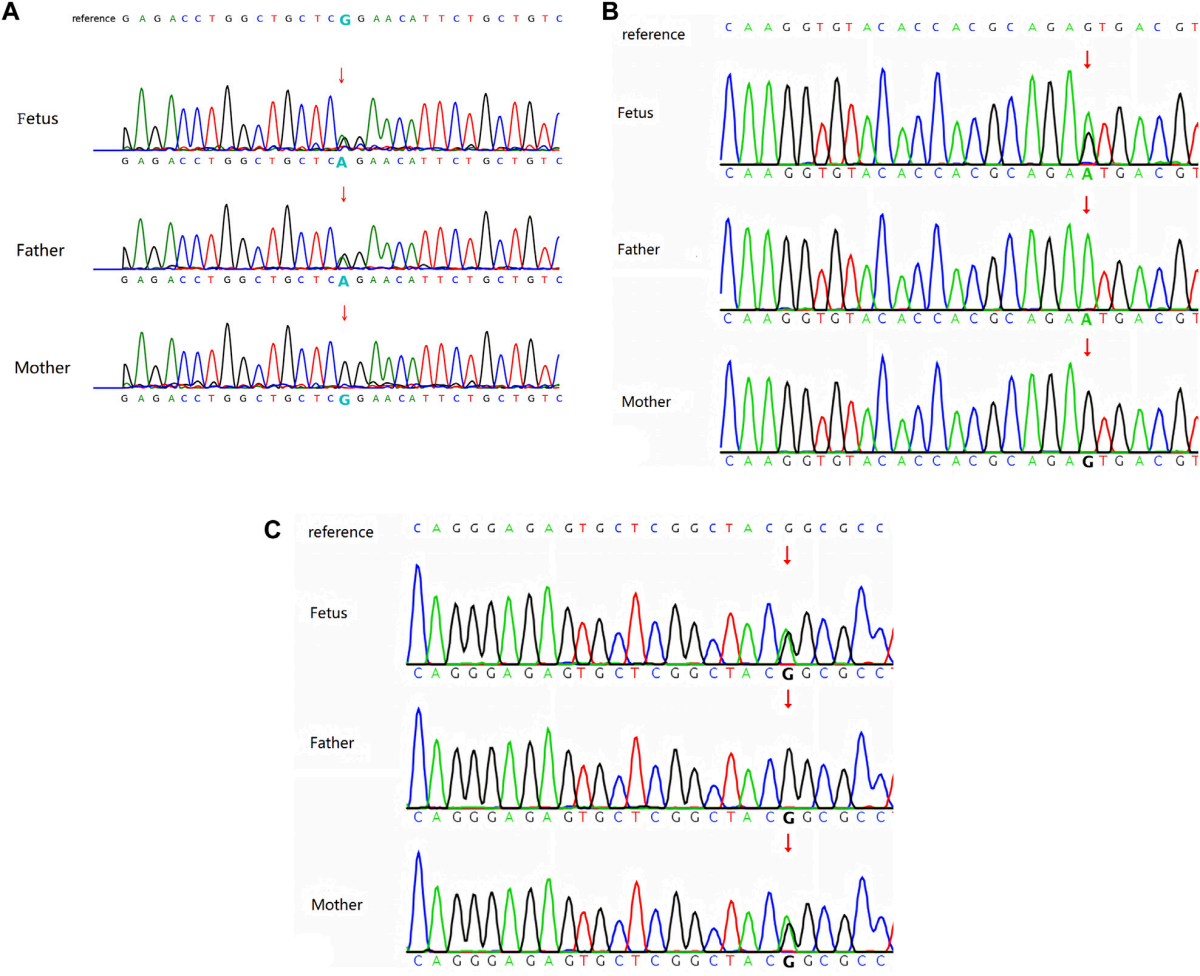
**(A)** Sanger sequencing result of family 1: heterozygous variant of c.3122G>A in the FLT4 gene. **(B)** Sanger sequencing result of family 2: heterozygous variant of c.3284G>A in the FLT4 gene. **(C)** Sanger sequencing result of family 3: heterozygous variant of c.2560G>A in the FLT4 gene.

A heterozygous variant of c.3122G>A in the *FLT4* gene was detected in the fetal sample of family 1 using WES, and Sanger sequencing revealed a heterozygous variant of c.3122G>A in the *FLT4* gene of the father, whereas the gene of the mother was wild type (see Figure 2A). This locus was rated as likely pathogenic according to the ACMG mutation rating guideline (PM1+PM2_Supporting + PP3+PP1_strong).

PM1: The variant was located in the protein kinase domain| protein kinase-like domain| serine–threonine/ tyrosine–protein kinase catalytic domain| tyrosine–protein kinase catalytic domain.
PM2_Supporting: The variant was not identified in the Exome Aggregation Consortium (ExAC), the Genome Aggregation Database (gnomAD), and the 1000 Genomes Project (1000genomes).
PP3: The variant was predicted using multiple computational methods to likely have a deleterious impact on the gene or gene product.
PP1_strong: The variant co-segregates with the disease in the pedigree [PMID: 12960217].

A heterozygous variant of c.3284G>A in the *FLT4* gene was detected in the fetal sample of family 2 using WES, and Sanger sequencing revealed a homozygous variant of c.3284G>A in the *FLT4* gene of the father, whereas the gene of the mother was wild type (see Figure 2B). This locus was rated as likely pathogenic according to the ACMG mutation rating guideline (PM1_Supporting + PM2_Supporting + PP3_Moderate + PP1+PP4).

PM1_Supporting: The MCR missense OE score of this variant is 0.30.
PM2_Supporting: The variant was not identified in the Exome Aggregation Consortium (ExAC), the Genome Aggregation Database (gnomAD), and the 1000 Genomes Project (1000genomes).
PP3_Moderate: The variant was predicted by REVEL to have a deleterious impact on the gene or gene product (score: 0.810).
PP1: The variant co-segregates with the disease in the pedigree of this case.
PP4: The disease associated with this variant is consistent with the phenotypes of the fetus and the father.

A heterozygous variant of c.2560G>A in the *FLT4* gene was detected in the fetal sample of family 3 using WES, and Sanger sequencing revealed a heterozygous variant of c.2560G>A in the *FLT4* gene of the mother, whereas the gene of the father was wild type (see Figure 2C). This locus was rated as likely pathogenic according to the ACMG mutation rating guideline (PP1_Strong + PP3+PS4_Supporting + PM2_Supporting).

PP1_Strong: The variant has been reported in the literature that it has co-segregated with the disease in at least five meioses across two independent pedigrees [PMID: 12960217].
PP3: The variant was predicted by REVEL to have a deleterious impact on the gene or gene product; the variant was predicted by Spidex to likely have an impact on splicing.
PS4_Supporting: The variant has been reported in a total of two probands with lymphedema in the literature [PMID: 12960217].
PM2_Supporting: The variant was not identified in the Exome Aggregation Consortium (ExAC), the Genome Aggregation Database (gnomAD), and the 1000 Genomes Project (1000genomes), and this known variant is rated as DM in the HGMD database [PMID: 29906362; 12960217].

### 3.4 Pregnancy outcome follow-up

**FIGURE 3:**
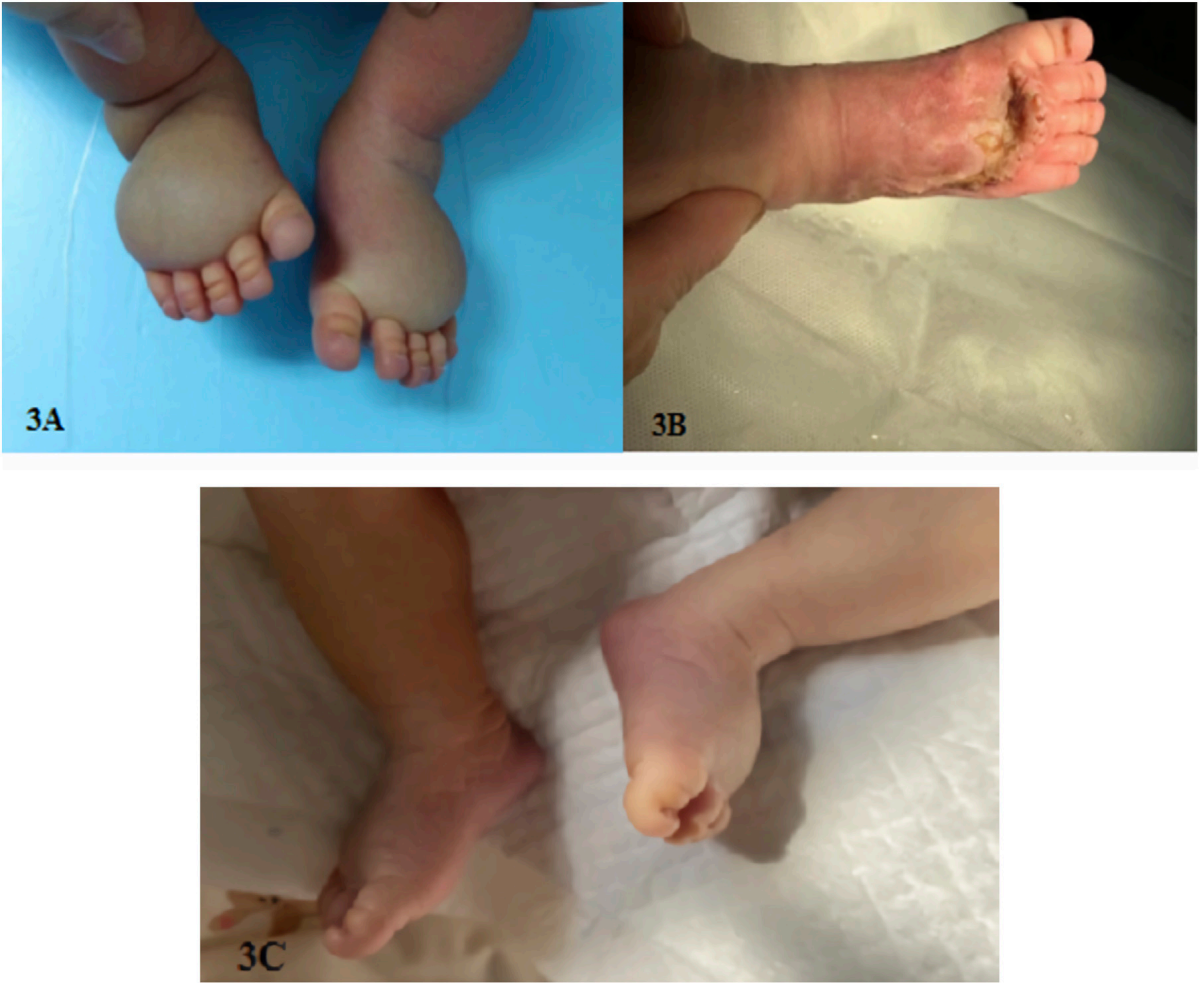
**(A)** Foot dorsum edema in family 1 newborn, **(B)** improved edema in family 1 newborn after surgery, and **(C)** mild edema on the dorsum of both feet in family 2 newborn.

Family 1 requested to continue the pregnancy after adequate genetic counseling. The on-going ultrasound monitoring during pregnancy showed a trend of aggravated edema in the feet dorsum of the fetus. The fetus was delivered naturally at full-term, and significant edema was observed in the dorsum of both feet after birth (see Figure 3A). Surgical treatment was performed at the age of 1 month, and the edema improved after surgery (see Figure 3B). Now, the infant is over 4 years old, with mild edema in the toes.

Family 2 also requested to continue the pregnancy after adequate genetic counseling. The continuous ultrasound monitoring during pregnancy showed a trend of relieved edema in the feet dorsum of the fetus. The fetus was born naturally at full-term, and mild edema was observed in the dorsum of both feet after birth (see Figure 3C). No medical intervention was performed, and the infant is now over 2 years old with deep skin folds at the base of both feet toes and dorsum skins.

Family 3 requested to terminate the pregnancy after adequate genetic counseling. The pregnancy was terminated at 24 WG. Although significant edema was observed in the dorsum of both feet in the induced abortion, the parents refused further autopsy.

## 4 Discussion

PCL caused by *FLT4* gene mutations is mainly manifested as dorsalis pedis edema before, during, or shortly after birth. This disease is caused by underdeveloped local lymphatic vessels, which then leads to lymphatic fluid retention in tissue spaces and finally causes edema (Zhang et al., 2019). Other positive characteristics include scrotal effusion, toenail protrusion, and male urethral abnormalities (Pauliina et al., 2020). PCL is most often diagnosed postnatally; therefore, there are few reports on the prenatal and fetal phenotypes of PCL. Ying et al. (2020) reported a case of PCL diagnosed during the neonatal period, characterized by a fetal phenotype of right-sided pleural effusion and combined postpartum pericardial effusion, pleural effusion, and edema in both lower limbs. Zeng et al. (2024) reported a case of PCL diagnosed during fetal development. The fetal ultrasound showed thickening of the nuchal translucency by 3.6 mm, with pleural and peritoneal effusion. However, the main fetal imaging manifestations of all the three families in this study were only thickening of subcutaneous tissues in both lower limbs and feet dorsum. Gordon et al. (2013) indicated clinical heterogeneity of the disease. Based on previous literature reports and the results of this study, PCL caused by *FLT4* gene mutations can present with phenotypes such as lymphedema in lower limbs and feet dorsum, pleural effusion, abdominal effusion, and thickening of the nuchal translucency during fetal development. Among them, lymphedema in bilateral lower legs and feet dorsum are characteristic clinical phenotypes of this disease during fetal development, which have important implications for early diagnosis of this disease.

The *FLT4* gene is located on chromosome 5q35 (Daniel-Spiegel et al., 2005) and is the only known gene associated with PCL disease. It contains 31 exons, with the coding protein VEGFR-3. The extracellular segment of VEGFR-3 can bind to ligands, further activating the intracellular segment containing two tyrosine kinase domains, initiating downstream signaling transduction pathway, and regulating the proliferation and development of lymphatic endothelial cells (Monaghan et al., 2021). It has been reported that a missense mutation in the *FLT4* gene can generate mutant receptors that form heterodimers with wild-type receptors on the cell membrane, causing a loss of cross-autophosphorylation, failure to activate intracellular signal transduction, and ultimately impaired the function of lymphatic endothelial cells (Liang and Gao, 2023; Liu and Gao, 2021).

Thus far, there are 60 reported *FLT4* gene variants associated with PCL disease, most of which are concentrated in tyrosine kinase domain 1 or 2 (TK1 or TK2), corresponding to exons 17–20 and 22–26, respectively (Gordon et al., 2013). Liu et al. (Monaghan et al., 2024) reported that two new variants in the extracellular immunoglobulin domain are also associated with PCL pathogenesis. Moreover, many previous studies (Monaghan et al., 2024; Zhang et al., 2024; Page et al., 2019) have suggested a close association between *FLT4* gene immunoglobulin domain variations and the occurrence of congenital heart disease (Tetralogy of Fallot). In this study, *FLT4* gene variation sites in three families occurred in exons 18, 23, and 24, respectively, of the tyrosine kinase domain. Among them, the heterozygous variant of c.3122G>A and the heterozygous variant of c.2560G>A have been reported in postpartum PCL infants (Evans et al., 2003) but never reported in fetus, and there have been no report of postpartum infants with the heterozygous variant of c.3284G>A. This variant is predicted by REVEL to have a deleterious impact on the gene and gene products. Combined with the phenotype of the proband’s father and other affected relatives, this evidence suggests that the variant is likely associated with the development of PCL. Based on the literature and the cases in this study, it is considered that the variation in the tyrosine kinase domain of the *FLT4* gene is the main genetic cause of PCL.

*FLT4* gene-related PCL is mostly inherited in an autosomal dominant pattern, also with autosomal recessive inheritance pattern reported (Melikhan-Revzin et al., 2015; Xiang et al., 2023). In this study, the fetal *FLT4* gene mutations are originated from either the father or the mother in all three families and consistent with autosomal dominant inheritance characteristics. The newborn from family 1 was found to have severe edema in both feet after birth and underwent surgical treatment at the age of 1 month; although the father had mild foot dorsum edema at birth, the symptoms of foot edema gradually subsided as he grew older. The degree of edema in the fetus from family 2 gradually decreased with increasing gestational age during pregnancy; the father has a homozygous variant of c.3284G>A in the *FLT4* gene, has had thickening and swelling on the dorsum of both feet since childhood, and has not fully recovered; his grandfather and his paternal aunt all showed unilateral thickening and swelling in the foot dorsum skin in adulthood. The fetus from family 3 developed lower limb edema during pregnancy, but the mother has not experienced any related clinical symptoms since childhood. As there is significant heterogeneity in the clinical manifestations of PCL, lower limb edema in severe cases can last for life, whereas some patients exhibit no obvious abnormal symptoms, and in some patients, the symptoms can self-alleviate. PCL rarely causes serious complications, with only occasional cases reported with complications such as cellulitis of dorsum and toes, along with severe pleural and peritoneal effusion (Gezginc et al., 2012). Currently, there is no specific therapy for PCL. The conservative therapies have certain effects on preventing lymphedema formation and improving mild lymphedema. For symptoms such as severe edema and later skin fibrosis, the surgical intervention is necessary (Guidelines for Hemangioma and Vascular Malformation Diagnosis and Treatment, 2024). Guo et al. (2020) reported a case of PCL discovered during fetal development; with twice interventional therapy after birth, the bilateral dorsum edema was improved. This is similar with the surgical prognosis of the infant from family 1 in this study. Based on previous literature reports and the cases in this study, with increasing gestational ages, the lymphatic circulation pathway can self-regulate and repair in fetuses, and some cases could naturally improve. Therefore, under the premise of adequate communication with the parents, proper genetic counseling, and strengthened monitoring during pregnancy, a personalized treatment plan can be developed based on the actual situation after the fetus is born.

The fetal phenotype analysis is an important component of diagnostic evaluation when suspecting fetal genetic diseases. Therefore, analyzing the fetal phenotype is crucial for guiding differential diagnosis and detection choice (Shear et al., 2025). In conclusion, the *FLT4* gene mutation is a key genetic factor leading to PCL. Accurately identifying this gene mutation during fetal development is of great significance for improving prenatal diagnosis accuracy, refining the genetic counseling system, optimizing pregnancy decision-making, and developing early clinical intervention strategies.

## Data Availability

All relevant data is contained within the article. The original contributions/raw data cannot be presented in the study or public repositories due to the restriction of the national Human Genetic Resource regulations. Any further inquiries shall be directed to the corresponding author.
